# Central Aspects of Pain in Rheumatoid Arthritis (CAP-RA): protocol for a prospective observational study

**DOI:** 10.1186/s41927-021-00187-2

**Published:** 2021-06-24

**Authors:** Onosi S. Ifesemen, Daniel F. McWilliams, Eamonn Ferguson, Richard Wakefield, Kehinde Akin-Akinyosoye, Deborah Wilson, Dorothy Platts, Susan Ledbury, David A. Walsh

**Affiliations:** 1 Division of Rheumatology, Orthopaedics and Dermatology, School of Medicine, University of Nottingham, Nottingham, UK; 2 Pain Centre Versus Arthritis, University of Nottingham, Nottingham, UK; 3 NIHR Nottingham Biomedical Research Centre, Nottingham University Hospitals NHS Trust, Nottingham, UK; 4 School of Psychology, University of Nottingham, Nottingham, UK; 5 Leeds Institute of Rheumatic and Musculoskeletal Medicine and NIHR Leeds Biomedical Research Centre, University of Leeds, and Leeds NHS Teaching Hospitals Trust, Leeds, UK; 6 Centre for Epidemiology Versus Arthritis, Centre for Musculoskeletal Research, Division of Musculoskeletal and Dermatological Sciences, School of Biological Sciences, Faculty of Biology, Medicine and Health, University of Manchester, Manchester, UK; 7 Rheumatology, Sherwood Forest Hospital NHS Foundation Trust, Sutton-in -Ashfield, Nottinghamshire, UK; 8 Independent Consultant, Nottingham, United Kingdom

**Keywords:** Rheumatoid arthritis, Pain, Central sensitization, Fatigue, Inflammation

## Abstract

**Background:**

Pain and fatigue are persistent problems in people with rheumatoid arthritis. Central sensitisation (CS) may contribute to pain and fatigue, even when treatment has controlled inflammatory disease. This study aims to validate a self-report 8-item questionnaire, the Central Aspects of Pain in Rheumatoid Arthritis (CAP-RA) questionnaire, developed to measure central pain mechanisms in RA, and to predict patient outcomes and response to treatment. A secondary objective is to explore mechanisms linking CS, pain and fatigue in people with RA.

**Methods/design:**

This is a prospective observational cohort study recruiting 250 adults with active RA in secondary care. The CAP-RA questionnaire, demographic data, medical history, and patient reported outcome measures (PROMs) of traits associated with central sensitization will be collected using validated questionnaires. Quantitative sensory testing modalities of pressure pain detection thresholds, temporal summation and conditioned pain modulation will be indices of central sensitization, and blood markers, swollen joints and ultrasound scans will be indices of inflammation. Primary data collection will be at baseline and 12 weeks. The test-retest reliability of CAP-RA questionnaire will be determined 1 week after the baseline visit. Pain and fatigue data will be collected weekly via text messages for 12 weeks. CAP-RA psychometric properties, and predictive validity for outcomes at 3 months will be evaluated.

**Discussion:**

This study will validate a simple self-report questionnaire against psychophysical indices of central sensitization and patient reported outcome measures of traits associated with CS in a population of individuals with active RA. The application of this instrument in the clinical environment could provide a mechanism-based stratification tool to facilitate the provision of targeted therapy to individuals with pain and fatigue in RA, alongside treatments that target joint inflammation.

**Trial registration:**

Clinicaltrials.gov NCT04515589. Date of registration 17 August 2020.

**Supplementary Information:**

The online version contains supplementary material available at 10.1186/s41927-021-00187-2.

## Background

Rheumatoid Arthritis is a (RA) chronic autoimmune inflammatory disease affecting hands, feet and other joints. RA typically presents with pain, joint stiffness, and fatigue. It may be associated with extra-articular inflammation affecting, for example, lungs or eyes [1]. RA runs a relapsing and remitting course through the lifetime of an individual. There is currently no cure, but inflammatory disease can usually be controlled using conventional or biologic Disease Modifying Anti-Rheumatic Drugs (DMARDs). New treatment regimens incorporating early intensive DMARD treatment have contributed to improving quality of life of people with RA.

DMARDs produce and sustain remission of inflammatory disease for many people, but, even then, persistent pain and fatigue can remain major problems. On average, pain incompletely improves after DMARD therapy, with 80% of people who considered their disease to be well controlled continuing to report pain [2]. The prevalence of clinically important pain among people with sustained RA disease remission longer than 1 year is about 12% [3]. About 40% of patients with well controlled inflammatory disease reported persistent fatigue [4, 5].

Pain and fatigue in RA arise from complex and overlapping mechanisms involving inflammation, structural joint changes, central and peripheral pain processing, as well as psychosocial factors, health beliefs and illness perceptions [6, 7]. In RA, pain results from interactions between multiple mechanisms in the peripheral and central nervous systems. Individuals with RA pain describe it as aching or gnawing suggesting a nociceptive pain, and some describe it as ‘shooting’ or ‘burning’, which is characteristic of neuropathic pain [6]. RA pain may have nociplastic features, described as widespread, and without evidence of commensurate tissue or nerve damage [8]. Nociplastic pain may be due to central sensitization [9, 10]. Central sensitization is a phenomenon characterised by increased responsiveness of nociceptive neurons in the central nervous system to their normal or subthreshold afferent input. CS may be associated with fatigue independent of pain [11], although people with Chronic Fatigue Syndrome often experience widespread pain [12, 13], and people with fibromyalgia experience chronic fatigue.

Central mechanisms are thought to drive pain and fatigue in the presence of well controlled inflammatory disease. The disease activity score in 28 joints (DAS28) is a specific quantitative clinical index to assess and monitor disease activity in RA [14] and CS may increase patient-reported components of DAS28 - tender joint count (TJC) and visual analogue scores (VAS), worsening the DAS28 score in the absence of concordant inflammation [15]. In these circumstances misclassification of active disease could result in increased use of DMARDs, exposing people to the risk of adverse events without real prospect of benefit [16]. Too narrow a focus on suppressing inflammation can displace the introduction of adjunctive treatments that might reduce central sensitisation and improve long-term pain and fatigue in RA.

Current RA treatment guidelines adopt a ‘one size fits all’ approach to pain management, mainly targeted at nociceptive pain [17]. However, different pain mechanisms represent different targets for pain management and their measurement might permit stratified or precision treatment delivery to alleviate pain in people with RA. Furthermore, available evidence describes a heterogenous RA population with varying prognosis and treatment responses in terms of disease activity, pain, fatigue, functional limitations, and psychological distress [18–22]. Distinct pain mechanisms as well as heterogenous symptom response presents an opportunity for targeted interventions for symptom control e.g. people with persistent pain due to resistant joint inflammation might benefit from a change in DMARD strategy, whereas those with pain driven by central mechanisms might better benefit from treatments that target the central nervous system.

Several research studies have demonstrated an association between central pain mechanisms and persistent and severe pain in musculoskeletal conditions [23–26] A stratification tool which identifies people with central sensitisation could improve the treatment of RA pain. Central pain mechanisms can be assessed using a variety of methods, including quantitative sensory testing (QST) [27–29] and self-report questionnaires [23, 30].QST includes several modalities that assess different aspects of central pain processing [27]. Although QST shows promise as a stratification tool, its application in clinical settings is limited by logistic issues such as requiring face to face contact with a trained practitioner and test duration [28, 31]. Questionnaires permit economical assessment with relative ease and are suitable for use with large populations and in busy clinical settings. Questionnaires that can assess central pain mechanisms should, therefore, be more amenable than QST for standard applications in clinics and in research.

A single page 8 item questionnaire, Central Aspects of Pain in the knee (CAP-knee) was designed and validated as a measurement and classification tool for central pain augmentation in people with knee pain. This self-report tool measures 8 characteristics strongly associated with central pain mechanisms and predicts 1-year pain outcomes in people with knee pain [23]. The Central Aspects of Pain in RA (CAP-RA) is a minor adaptation of the CAP-knee questionnaire for use in a RA population. This study aims to further develop and validate the new self-report 8 item CAP-RA questionnaire for measuring central pain mechanisms in RA.

### Objectives

The primary objective is to:

1. to optimise and establish the psychometric properties of CAP-RA and

Secondary objectives are to:

1. measure the ability of CAP-RA to identify people with pain that is augmented by central mechanisms, are destined to have poor pain outcomes despite modern therapy directed at joint inflammation.
2. Investigate factors associated with worse RA pain or fatigue at baseline and 3 months follow up
3. Compare the performance of CAP-RA to other predictors of pain outcomes
4. Derive CAP-RA scoring recommendations for stratification in clinical trials and in clinical practice
5. Examine associations between pain, central sensitization and fatigue in RA.
6. Examine the short-term course of pain and fatigue in RA

## Methods/design

### Study design and setting

CAP-RA is an observational prospective study based in secondary care. Adults with RA who meet the study inclusion criteria will be recruited through secondary care rheumatology services (Sherwood Forest Hospitals NHS Foundation Trust; SFH). Participants will undergo clinical assessments, blood tests, ultrasound scans and complete the study questionnaire booklet at baseline, and 12 weeks later. A consecutive sample of participants will in addition complete the CAP-RA questionnaire 1 week after the baseline visit.

### Ethical considerations and study sponsor

The study submitted to the national integrated research application system (IRAS) and was reviewed and approved by the North of Scotland Research Ethics Committee (1) (reference no: 20/NS/0036) and will be performed in accordance with the UK policy framework for Health and Social Care Research, 2018, principles of good clinical practice and Helsinki Declaration. A SPIRIT 2013 statement checklist [32] is included in supplement 1. The CAP-RA study sponsor is the University of Nottingham (which supervises data management procedures, ethics amendment processes/dissemination, and study stopping guidelines) and the current protocol is version 1.2,18/08/2020. Samples of all study related documents including consent forms and participant communication are also included in supplement 2 and 3.

Informed consent will be sought and confirmed through a signed consent form at the start of the first study visit before commencing study procedures. Participants would be explicitly informed that partaking in the study would not affect the participant’s entitlements, treatment, or care during the study or in the future; and would not require or preclude participation in other research projects. Details about how to report study concerns or medical issues are also provided.

### Public and patient involvement and engagement

People with RA and members of the public were involved in the conceptualization and design of the study. The original CAP-knee questionnaire was developed in conjunction with patients and members of the general public. The minor wording adjustments to the CAP-knee questionnaire to suit a RA population were undertaken jointly by researchers (OSI, KAA, DMcW) and patients through face to face meetings and by email. In addition, the CAP-RA study steering committee charged with overall management of the study comprises researchers and people with RA. The steering committee is charged with monitoring all aspects of study and ensuring that study conduct adheres to the study protocol and timescales.

Participants will be updated on research progress through newsletters. Research findings will be disseminated though multiple routes, such as spoken presentations, conference abstracts, online and in academic publications.

### Participants

Inclusion criteria: All of the following

- Adults aged 18 years and above
- Satisfy the European League Against Rheumatism (EULAR) criteria for RA [33]
- Have active RA, as defined as DAS28 ≥ 3.2 at the baseline visit
- Pain levels > 3 on a 0 to 10 numerical rating scale where 0 = no pain and 10 = worst imaginable pain.
- Ability to give informed consent

Exclusion criteria: Any of the following -

- Unable to give informed consent
- Insufficient understanding of spoken or written English to comply with the requirements of the study protocol.
- Unable or unlikely to complete the proposed 12-week study follow up (e.g moving house, terminal diagnosis, current or planned pregnancy).
- Active co-morbidity (e.g uncontrolled diabetes mellitus, cancer, infection) requiring changes in medical treatment at baseline.
- Major active psychiatric condition (e.g major depression)
- Inability to meet the requirements of clinical assessments

### Recruitment

Participants will be recruited from secondary care. Outpatient records will be screened by a member of the clinical care team, and eligible participants will be contacted by letter and invited to join the study. The study will be publicised in posters placed in public areas within clinics/hospitals. Additionally, members of the research team will recruit participants via face:face discussions in Rheumatology outpatient clinics in accordance with local policies.

### Study regimen

**Fig. 1:**
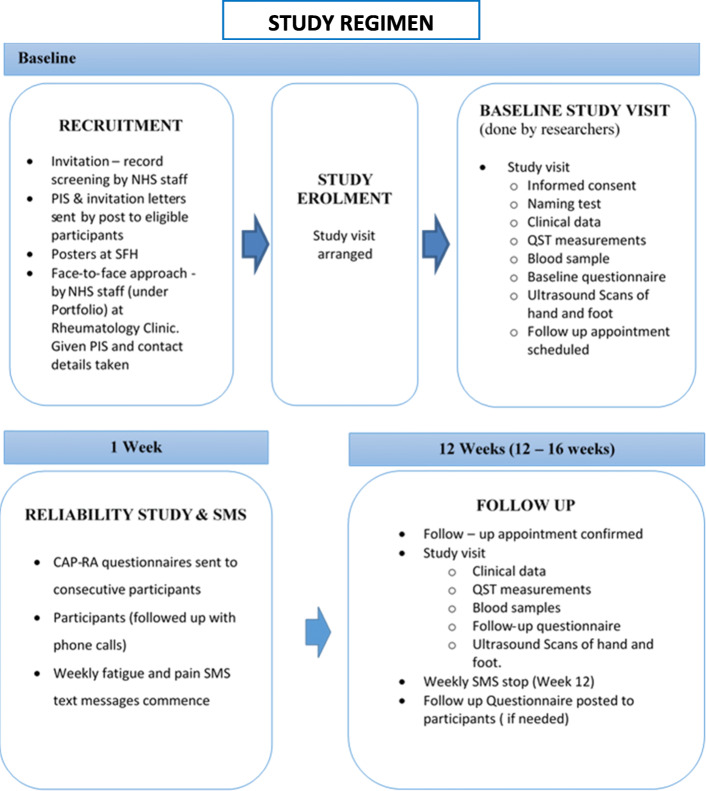
Schematic diagram of CAP-RA study regimen

The baseline and 12-week study visits will be held in a private clinic room. At the baseline visit, the participant’s eligibility will be verified, and informed consent will be obtained. The baseline and 12-week study assessments will have identical data collection protocols. A time window of 12–16 weeks will be acceptable for the follow up assessment. At 1 week after baseline assessment, a subset of 80 people will be invited to complete and return a postal questionnaire consisting of only the 8-item CAP-RA questionnaire to examine test-retest reliability. For the study duration, participants may opt-in to receive and respond to SMS text messages asking about weekly pain and fatigue levels. The study regimen is described in Fig. 1.

### Data collection

#### Questionnaire

The study questionnaire booklet was developed using validated instruments as well as the newly designed CAP-RA questionnaire.

RA pain will be measured using 0–10 numerical rating scale and fatigue [34], using the Bristol RA Fatigue Scale (BRAFS) multidimensional questionnaire [35].

Traits associated with central pain mechanisms will be assessed using the CAP-RA questionnaire and the Central Sensitization Inventory short form-9(CSI-9) [36, 37]. Information on neuropathic-like pain will be collected by a modified painDETECT questionnaire [38]. The waveform and pain intensity questions were removed as these are not included in the neuropathic pain score, and questions were reworded as statements for easy readability. Additionally, the questionnaire will utilise, the pain catastrophising scale (PSC) [39], and the Bristol RA Fatigue Scale (BRAFS) multi-dimensional questionnaire [35]. Depression and anxiety will be measured using the Hospital Anxiety and Depression Scale (HADS) [40]; functional ability,; and sleep, by the Athens Insomnia Scale (ASC) [41]. Cognitive function will be measured using the Cognitive Failures Questionnaire (CFQ) [42].

The questionnaire will also address conditions, treatments and outcomes that are associated with central pain mechanisms and fatigue. The presence of fibromyalgia will be classified using pain distribution from a self-completed whole-body manikin, to derive the Widespread Pain Index (WPI), and Symptom Severity Score (SSS) [43, 44]. Disability and physical activity will be assessed with the Health Assessment Questionnaire (HAQ) [45] and the short International Physical Activity Questionnaires (IPAQ) [46] respectively. Demographic characteristics will be recorded, and comorbidities required to calculate the Rheumatic Disease Co-Morbidity Index (RDCI) will be assessed [47]. A detailed medication history will be collected, including common medications used to manage RA, common pain medications e.g. nonsteroidal anti-inflammatory drugs (NSAIDs), paracetamol.

#### Clinical assessment

During the study visits, participants will undergo the following data collection procedures

i. Study questionnaire will be administered to all participants. The questionnaire booklet will be self-completed by the participant.
ii. A 1 min cognition test will be performed where the participant names as many animals as possible from memory [48].
iii. DAS28: Tender and Swollen joint count assessment: 28 joints, the 10 metacarpophalangeal (MCP), 10 proximal interphalangeal (PIP), 2 wrist, 2 elbow, 2 shoulder and 2 knee joints will be examined for tenderness and swelling [49]. Patient Global Assessment (PGA) of disease activity will be assessed using a visual analogue scale. DAS28-ESR will be estimated using the following formula

Equation 1: DAS28 ESR Calculation [50]

$$ DAS28=0.56\times \sqrt{TJC}+0.28\times \sqrt{SJC}+0.70\times \ln ESR+0.014\times PGA $$

iv. Quantitative sensory testing will be conducted using Pressure Pain detection Threshold (PPT), Temporal Summation (TS) and Conditioned Pain Modulation (CPM) tests. PPT involves the assessment of mechanical pressure pain thresholds using a handheld computerised pressure algometer (Medoc Algomed – Computerised Pressure Algometer). The algometer consists of a rod with a 1 cm diameter circular end and is applied perpendicular to the test site. Pressure is applied through the algometer at a standardised rate until the participant presses a button indicating a change in sensation from pressure to pain, when the algometer is immediately removed and the pressure reading recorded. Three measurements will be collected at each site, with a short rest between measurements. Participants will be familiarized with the procedure by performing it on a different test site before commencing the test. PPT will be conducted on three anatomical sites, the body of the brachioradialis muscle of the non-dominant forearm, the medial tibiofemoral joint line, and the body of the tibialis anterior muscle of the dominant side [51].
  Temporal summation (TS) tests the nervous systems responsiveness to repeated noxious stimuli. It will be conducted using a supra-threshold pinprick stimulator (256mN pinprick), which is applied repeatedly to a single point on the skin over the patella ligament, in a single test and on a train of 10 repetitions, with intervals of 1 s in between repetitions. The participants will be asked to indicate the intensity of pain on a 0–100 visual analogue scale (where 0 represents no pain and 100, the most intense pain imaginable) for the single test, and the average pain intensity for the series. The difference between the 2 self-reports will be used to measure TS.
  Conditioned Pain Modulation (CPM) is conducted using the PPT test (test stimulus). The test stimulus is applied to the body of the tibialis anterior muscle contralateral to the participant’s dominant hand before and after induction of a painful stimulus at a remote anatomical site. This conditioning stimulus is achieved by applying a blood pressure cuff to the dominant arm and inflating until the radial pulse can no longer be palpated. The participant may be asked to perform hand exercises (squeezing a stress ball) to induce exercise induced ischaemic pain or discomfort. As soon as the pain or discomfort in the forearm is rated as 4/10 by the participant, they stop squeezing the ball and the second PPT test is performed. Only one CPM test will be performed. CPM is calculated as [52]:

Equation 2: CPM Calculation

$$ CPM= PPTpost- PPTpre $$

xxii. Joint inflammation (synovitis and tenosynovitis) will be assessed by ultrasound scans of the hands and feet in both grayscale (GS) and power Doppler (PD) modes. Examinations will be conducted according to the EULAR guidelines for MSK ultrasound scans in rheumatology [53]. Joint inflammation will be assessed using the OMERACT US group scoring criteria [54, 55].
xxiii. Blood samples will be obtained for inflammatory biomarker (Erythrocyte Sedimentation Rate (ESR), and C-Reactive Protein (CRP) assays, and samples may be stored for additional biomarker analyses.

#### Additional data collection

The CAP-RA questionnaire will, in addition, be mailed for completion by 80 consecutive participants 1 week after the baseline visit, to assess test-retest reliability.

The course of fatigue and pain will be assessed by sending participants weekly SMS text messages at approximately the same time each week, with the question “On a scale of 0 – 10, with 0 indicating no pain, and 10 indicating the worst imaginable pain, how would you rate your pain during the past week? Please respond to this message with a number. Thank you”. The equivalent question wording will be used to ask about weekly fatigue.

### Statistical analysis

The primary objective of the study is to determine the psychometric properties of the new CAP-RA questionnaire. The psychometric properties examined will include structural validity, construct hypothesis testing, internal consistency, reliability, measurement error and criterion validity, in accordance with Consensus-based Standards for the selection of health Measurement Instruments (COSMIN) guidelines [56–58].

Structural validity measures the degree to which the scores in a patient reported outcome measure reflect the dimensionality of the construct to be measured. Structural validity will be assessed using confirmatory factor analysis (CFA) and Rasch modelling. A suitable polytomous Rasch model will be selected [56]. The unidimensional and local independence assumptions will be examined and model fit parameters will be estimated [59]. The construct validity of the CAP-RA questionnaire, a measure of the degree to which scores are consistent with a hypothesis that is based on the assumption that the PROM validly measures the intended construct, will be investigated using confirmatory factor analysis. Appropriate fit statistics will be calculated [60]. Internal consistency, which is a measure of the degree of interrelatedness among items in the questionnaire will be estimated using Cronbach’s alpha, with a value greater than 0.70 signifying acceptable internal consistency [61].

Test-retest reliability will be estimated using the Intraclass Correlation Coefficient, with acceptable values ≥0.70 [62]. Measurement error will be estimated by the Standard Error of Measurement (SEM), Smallest Detectable Change (SDC) and the Limits of Agreement (LoA). Criterion validity will be a measure of the degree to which the instrument is an adequate reflection of central sensitisation.

The relationship between baseline CAP-RA scores and pain outcomes will be measured using regression analysis, with 12-week pain as the outcome variable and baseline characteristics as covariates.

Secondary objectives will be addressed using appropriate regression analysis. For example, the association between fatigue and central sensitization will be examined using linear or non-linear regression analysis, based on the distribution of the data, and the course of fatigue will be examined using linear or non-linear mixed effect models as indicated. CAP-RA questionnaire thresholds for delimiting high levels of central pain mechanisms will be derived through receiver operating curves plotted using dichotomised outcome measures, such as the different QST measurements [56, 63].

Analyses will be undertaken using appropriate statistical software. *P* values less than or equal to 0.05 will be considered statistically significant unless otherwise stated.

### Sample size considerations

The overall study sample size has been calculated to determine the structural validity of the instrument using Rasch analysis. Our Rasch model assumes that item calibrations are within ±½ logit from stable values, with a 99% confidence interval. This gives an overall sample size of between 108 and 243, best to poor targeting [64]. We expect 200 participants will complete the study, from a sample of 250 participants. This is in keeping with COSMIN recommendations for Rasch analysis [56–58].

For longitudinal analysis over 12 weeks, we predict that *n* = 200 people will complete both study visits. The power calculation for a linear multiple regression with CAP-RA plus 5 baseline covariates (each assumed to have a correlation coefficient of 0.15 with the 12-week pain) yielded a sample size of *n* = 164 for α = 0.05 and power = 0.95.

Seventy-seven participants are required for the reliability study to estimate an ICC of 0.8, with 2 ratings, alpha of 0.05, 80% power, and a lower limit ICC not less than 0.6. Sample size calculation was implemented in R using “ICC.sample.size package” [65, 66].

## Discussion

CAP-RA questionnaire is a brief questionnaire that encompasses traits associated with central pain mechanisms in one tool. This is a prospective study primarily aimed at validating CAP-RA to measure and ultimately stratify central pain mechanisms in people with RA. The study is based on the premise that persistent pain in RA, in some people is caused by central mechanisms and that targeted adjuvant treatment provided to this specific population would greatly improve health outcomes in these individuals. If translated into the clinic, CAP-RA could help to assign people with RA to their optimal treatment in a more efficient manner.

The CAP-RA questionnaire will undergo rigorous psychometric testing to establish it as a validated tool to measure central pain mechanisms in individuals with RA., Pain catastrophising, depression, anxiety, neuropathic pain, pain distribution, sleep disturbance, fatigue, and depression have been found to represent a single ‘central mechanisms of pain’ trait in a population with knee osteoarthritis [28]. This current study will establish if these traits also characterise central pain mechanisms are in another musculoskeletal disorder and might therefore have general validity across a range of conditions. This study is also designed to discover if other measures of central mechanisms are able to predict future pain or fatigue, and hence to validate our underlying mechanistic hypothesis. Previously validated instruments, such as the CSI-9, PainDETECT and QST will be tested as predictors of pain or fatigue in a similar way to CAP-RA. As inflammation, specifically synovitis, is a known cause of RA pain, we will directly measure this with US to ensure that it is documented with sufficient detail and that its contribution to pain and pain prognosis is included within our analyses [67]. We predict that associations between central mechanisms and RA pain are moderated by inflammation. The scenario where synovitis resolves or improves upon commencement of a new DMARD, but pain persists, is of importance to clinicians and patients alike.

The CAP-RA questionnaire will extend the repertoire of quick and self-administered tools specifically designed to identify people with specific pain mechanisms. The CSI was designed to identify and quantify symptoms experienced by people with diagnoses such as fibromyalgia that have been associated with central sensitization. It has demonstrated good psychometric properties in multiple patient populations, but does not consider traits of neuropathic pain [36], which we have previously found to be part of the Central Mechanisms Trait in people with knee pain [68]. The originator painDETECT questionnaire has also been used to imply central pain augmentation in people with RA [38], although this alone was inferior to the central mechanisms trait in predicting PPT in people with knee pain [23]. The Allodynia Symptom Checklist is mostly used in people who suffer with headaches and has not been validated in a population with MSK pain [69, 70]. Another recently developed 11-item self-report questionnaire including items assessing pain and somatic symptoms also has not been validated in a RA population [71].

### Limitations

Although a brief questionnaire may be acceptable to patients, and feasible in busy clinical practice or large epidemiological studies, its interpretation is inevitably limited by its self-reported nature, and even validated self-report instruments are prone to recall bias. Traits assessed with the CAP-RA questionnaire are however multifaceted and subjective precluding objective measurement. We attempt to address this by testing CAP-RA against QST, as a proxy for central pain mechanisms. However, there is no gold standard for measuring CS which is itself a multidimensional construct. We will use a brief QST procedure which has been used in other research studies to measure central pain mechanisms, comprising both static (PPT) and dynamic (TS and CPM) test modalities. Each QST modality might have different measurement properties and indicate different aspects of CS including spinal processing of nociceptive signals and descending modulation. Only two timepoints will be measured, and this study was not designed to measure the ability of CAP-RA to predict persistent pain in RA beyond 3 months. This study will be conducted within a restricted geographical region (within the East Midlands of the UK) with people who have active rheumatoid arthritis measured by DAS28. Our findings might have limited generalizability outside this population. More bias may also occur with the weekly SMS text messages, as subgroup of participants opting in to this section of the study may not be representative. However, usage of SMS texting has previously been shown to be widely used in older populations with arthritis [72]. This study is reported in line with SPIRIT guidelines [32] as guidelines for cohort study protocols are not fully developed [73].

## Conclusion

Pain and fatigue are persistent symptoms experienced by people with RA, even in well controlled inflammatory disease. Persistent pain and fatigue may be caused by mechanisms within the central nervous system linked to central sensitization. This study seeks to validate a simple self-reported questionnaire to identify central mechanisms in the clinical setting to facilitate the identification of individuals who may benefit from adjuvant treatments alongside those aiming to reduce joint inflammation.

## Supplementary Information

**Additional file 1: Supplement 1.** SPIRIT Checklist describing protocol items for clinical trials.

**Additional file 2: Supplement 2.** CAP-RA PIS final version 1.218082020. CAP-RA Participant Information Sheet, describing the study protocol to potential participants and members of the public in lay language.

**Additional file 3: Supplement 3.** CAP-RA consent form final version 1.423092020 CAP-RA study participant consent form.

## Data Availability

The datasets generated and/or analysed during the current study may be made publicly available following conclusion of ongoing research. Requests for data may be made at any time to the corresponding author.

## Abbreviations

ACR: American College of Rheumatology
CS: Central Sensitization
DAS28: Disease activity score in 28 joints
DMARDS: Disease Modifying Anti-Rheumatic Drugs
EULAR: European League Against Rheumatism
PROM: Patient Reported Outcome Measure
QST: Quantitative sensory testing
RA: Rheumatoid Arthritis
SFH: Sherwood Forest Hospitals NHS Foundation Trust
TJC: Tender joint count
VAS: Visual Analogue Scores
