# Supplementary material for: Central Aspects of Pain in Rheumatoid Arthritis (CAP-RA): protocol for a prospective observational study
Source: BMC Rheumatol. 2021 Jun 24;5:23. doi: 10.1186/s41927-021-00187-2 (PMC8223274; doi:10.1186/s41927-021-00187-2)
Supplement: Supplementary file 2 — Additional file 2: Supplement 2. CAP-RA PIS final version 1.218082020. CAP-RA Participant Information Sheet, describing the study protocol to potential participants and members of the public in lay language. [file 41927_2021_187_MOESM2_ESM.pdf]

Study Title: Central Aspects of Pain in Rheumatoid Arthritis (CAP-RA)
IRAS Project ID: 269143
Principal (Chief) Investigator and Study Doctor: Professor David Andrew Walsh, Consultant Rheumatologist Sherwood Forest Hospitals NHS Foundation Trust and Professor of Rheumatology, University of Nottingham.
Study Sponsor: University of Nottingham

Participant Information Sheet

You are being invited to take part in a research study by Sherwood Forest Hospitals NHS Foundation Trust and the University of Nottingham. The study is investigating pain in people with rheumatoid arthritis. Before you decide, it is important to understand why we are doing the study, how your information will be used, what the study will involve and the potential benefits, risks and possible discomforts.

If you are willing to take part, you will be told about the research study and asked to initial and sign a consent form and will be given a copy to keep. If you give consent, the study may start immediately.

Why is this study being done?

Pain and fatigue are serious problems, even in people whose rheumatoid arthritis is well treated. In our other research, we have found that joint swelling and inflammation are not the only causes of pain. The central nervous system (brain and spine) does not merely sense pain; it also contributes to pain severity and can stop pain getting better. When the central nervous system makes pain much worse, we call this central sensitisation. Fatigue can be a part of this. The central nervous system also can sometimes block pain and make it feel better. Pain can affect how we think, feel and concentrate. Through this study, we hope to find better ways to measure central sensitisation and pain, to help us to develop better treatments. Parts of this study contribute to a PhD project.

Who can take part?

We are interested in everyone who has rheumatoid arthritis, even if pain and fatigue are not big problems for you. We are interested in people who rate their pain as greater than 3 on a 0 to 10 scale, where 0 is 'no pain' and 10 is the 'worst imaginable pain'. We hope that about 250 people will take part in this research study.

What are we asking you to do?

If you are interested in participating in this study, we would like you to come to King's Mill Hospital for clinical assessment, to complete questionnaire booklets, have ultrasound scans of your hands and feet, and give blood samples. These will be arranged at dates and times convenient for you. We may send you a short questionnaire a week after your first assessment. 3 - 4 months after the first visit, we would like to invite you to another visit. You will be asked to make 2 visits to King's Mill Hospital in total.

At each visit, you will see a member of our research team who has been specially trained for this study. Each study visit should be finished in 2-3 hours, and will take place in a private clinic room.

During the study visit you will be asked to do the following things:-

- Answer questions to check if you are suitable for this study, talk about the study and give your consent to participate
- Discuss your medical records and information that could be relevant to arthritis, and fill in a booklet of questions about your pain, health, sleep, medication and arthritis. This will include our new CAP-RA questionnaire. These are called “Study Data”).
- Have your joints examined for arthritis.
- Do a 60 second memory test.
- Be clinically assessed to see how you feel pain. Any pain that you feel during this assessment will be brief and should not be severe.
    - A researcher will use a softheaded probe on your forearm, knee and leg to measure what is the lowest pressure that you feel as pain (pain detection threshold).
    - A researcher will assess your pain sensitivity by applying to your knee the tip of a specially manufactured pen.
    - A researcher will again assess your pain detection threshold with the soft-headed probe applied to your leg while your other arm is compressed by a blood pressure cuff. You will be asked to squeeze on a small soft ball (stress ball).
    - Have an ultrasound scan. A small handheld probe will be placed on and moved over the skin of your hand and foot. A lubricating gel is put on your skin to allow the probe to move smoothly. You shouldn't feel anything other than the touch of the probe on your skin, and the gel (which is often cold).
- Provide a blood sample (which could be up to 30 ml (6 tubes) or about 6 teaspoons) which we will use to help measure inflammation and other molecules relevant to arthritis. Your blood might be used for genetic tests in order to determine whether genetic makeup has any connection with disease.

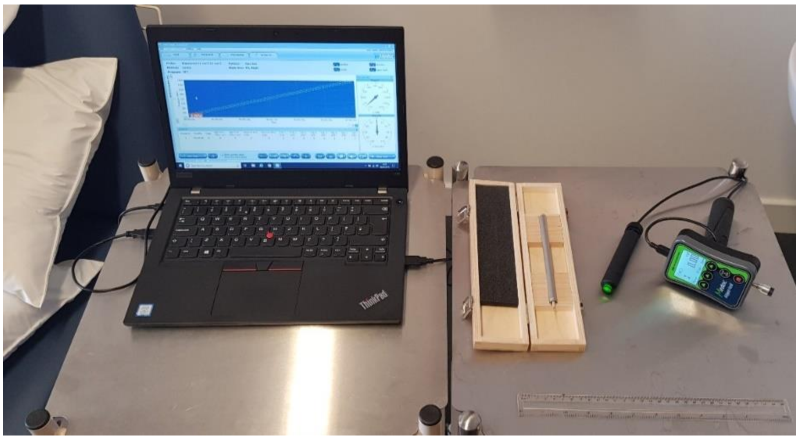

Pain assessment equipment

So that we can measure how much your answers might change, some people will be sent a second copy of the new CAP-RA questionnaire through the post a week after the study visit and asked to return the completed questionnaire in a pre-paid envelope.

We would also like to know how your fatigue and pain levels change from week to week. If you agree, you will receive 2 text messages each week for 12 weeks. The text messages will ask about your pain or fatigue levels during the past week on a scale of 0 – 10. You will be asked to reply to the text with a number. Text messages will be free of charge.

10 days before the date of your 2nd visit, you will receive a phone, text, email or letter from the research team to confirm your appointment. You may receive another message a day before the study to remind you of the appointment. If due to some unforeseen circumstances you are unable to attend the second visit, you may be sent a questionnaire by post, to return in a prepaid envelope. You may contact the research team at any time during the study if you wish to reschedule your appointment.

Researchers at the University of Nottingham might wish to contact you with further information or to invite you to contribute further to their research. Receiving further information will not commit you to joining any studies.

What are the benefits of my taking part?

It is not intended for you to have direct personal gain from your participation. You will not be paid for taking part in this study. Travel expenses will be offered for any visits incurred as a result of participation (on provision of receipts and up to a maximum of £20).
Taking part in this study means that you may possibly help other arthritis sufferers in the future. Being able to measure the changes that occur in people with painful arthritic joints could be used to develop new ways of treating arthritis.

What are the risks if I take part?

We are not anticipating any significant risks to participants in this study. We appreciate that you are giving up your valuable time and will try to minimise the inconvenience. You will experience some mild and brief pain during the pain assessment. Everyone experiences pain sensations differently so if you ask us to stop the procedure, for any reason, we will immediately stop.

What if there is a problem?

If you have a concern about any aspect of this study, you should ask to speak to the researchers who will do their best to answer your questions. The researchers' contact details are given at the end of this information sheet. If you remain unhappy and wish to complain formally, you can do this by contacting the King's Mill Hospital patient advice and liaison service(PALS) at 01623 672222, email : PET@sfh-tr.nhs.uk.

In the unlikely event that something does go wrong and you are harmed during the research and this is due to someone's negligence then you may have grounds for a legal action for compensation against the University of Nottingham but you may have to pay your legal costs. The normal National Health Service complaints mechanisms will still be available to you.

Taking part is entirely voluntary.

It is up to you to decide whether or not to take part. If you do decide to take part you will be given this information sheet and the consent form to sign and keep. If you decide to take part you are still free to withdraw at any time without giving any reason, and without your legal

rights being affected. If you choose not to take part, now or in the future, you will not be disadvantaged in any way, including your medical treatment and the care you are entitled to receive. You will be told if any new findings occur during the study that may affect your willingness to take part.
If you are no longer able to give consent, or withdraw from the study, we will no longer collect any information about you or from you. We will keep the information about you that we have already obtained, as we are not allowed to tamper with study records and this information may have already been used in some analyses and may still be used in the final study analyses. To safeguard your rights, we will use the minimum personally-identifiable information possible. We will assume that you are still willing to take part in the study if you send us completed questionnaires by post or attend follow up study visits.

What will happen to any samples I give?

Blood samples collected will be analysed for markers of inflammation. Clinically significant blood test results will be shared with your clinical care team. We would also like to seek your consent so that any remaining samples may be stored and used in possible future research – this is optional (please indicate on the consent form if you agree to this). The samples will be stored with a code unique to you and securely at the University of Nottingham under the University's Human Tissue Research Licence (no 12265). Some of these future studies may be carried out by researchers other than current team, who ran the first study, including researchers working for commercial companies. Any samples or data used will be anonymised, and you will not be identified in anyway. If you do not agree to this, any remaining samples will be disposed of in accordance with the Human Tissue Authority's codes of practice.

Please note that the link between you and the coded samples you have donated and the associated medical data will be kept as long as your samples are retained. The link to your study data will be kept for at least 7 years after the end of the study. After this time the study data will be fully anonymised, as the link will be broken and so your Study Data can no longer be traced back to you.

Will taking part be confidential?

We will follow ethical and legal practice and all information about you will be handled in confidence. If you join the study, we will use information collected from you and your medical records during the course of the research. This information will be kept **strictly confidential**, stored in a secure and locked office, and on a password protected database at the University of Nottingham. Under UK Data Protection laws the University is the Data Controller (legally responsible for the data security) and the Chief Investigator of this study (named above) is the Data Custodian (manages access to the data). This means we are responsible for looking after your information and using it properly. Your rights to access, change or move your information are limited as we need to manage your information in specific ways to comply with certain laws and for the research to be reliable and accurate. To safeguard your rights we will use the minimum personally – identifiable information possible.

You can find out more about how we use your information and to read our privacy notice at:

https://www.nottingham.ac.uk/utilities/privacy.aspx.

The data collected for the study will be looked at and stored by authorised persons from the University of Nottingham who are organising the research. They may also be looked at by authorised people from regulatory organisations to check that the study is being carried out correctly. All will have a duty of confidentiality to you as a research participant and we will do our best to meet this duty.

Where possible information about you which leaves the Hospital site will have your name and address removed and a unique code will be used so that you cannot be recognised from it, however sometimes we need to ensure that we can recognise you to link the research data with your medical records so in these instances we will need to know your name and date of birth. Your contact details will be held by the University for the duration of the study in order to arrange the study visits and send out questionnaires. If you agree to receive the text messages we will share your telephone number with the text messaging service, who will delete it at the end of the study.

Your contact information will be kept by the University of Nottingham for 15 years after the end of the study so that we are able to contact you about the findings of the study and possible follow-up studies (unless you advise us that you do not wish to be contacted). This information will be kept separately from the research data collected and only those who need to will have access to it. All other data (research data) will be kept securely for 7 years. After this time your data will be anonymised and retained. During this time all precautions will be taken by all those involved to maintain your confidentiality, only members of the research team given permission by the data custodian will have access to your personal data.

In accordance with the University of Nottingham's, the Government's and our funders' policies we may share our research data with researchers in other Universities and organisations, including those in other countries, for research in health and social care. Sharing research data is important to allow peer scrutiny, re-use (and therefore avoiding duplication of research) and to understand the bigger picture in particular areas of research. Data sharing in this way is usually anonymised (so that you could not be identified) but if we need to share identifiable information we will seek your consent for this and ensure it is secure. You will be made aware then if the data is to be shared with countries whose data protection laws differ to those of the UK and how we will protect your confidentiality.

Although what you say to us is confidential, should you disclose anything to us which we feel puts you or anyone else at any risk, we may feel it necessary to report this to the appropriate persons.

What will happen to the results of this study?

The results of the study may be published in medical literature and made more widely available, for example through our website (http://www.nottingham.ac.uk/paincentre), but your identity will not be revealed.

Who is organising and funding this study?

This study is organised and carried out by staff from the University of Nottingham and Sherwood Forest Hospitals NHS Foundation Trust. It is funded by Pfizer Ltd, Versus Arthritis and the National Institute for Health Research through grants to the University of Nottingham. The funders have no influence over the conduct of this study.

Who has reviewed this study?

All human research in the University of Nottingham is reviewed by independent groups of people, called a Research Ethics Committee to protect your interest. All materials associated with this study have been reviewed and approved by the North of Scotland (2) NHS Research Ethics Committee as well as the Health Research Authority.

Who can I contact if I have any questions about this study?

If you have any further questions about this study, or wish to contact the Study Sponsor, please discuss this in the first instance with: **Professor David Walsh**, Principal (Chief) Investigator and Study Doctor**. Telephone :**01158231676, **Email:** msk-recruitment@nottingham.ac.uk

Thank you for taking the time to read this information sheet
