# Supplementary material for: Central Aspects of Pain in Rheumatoid Arthritis (CAP-RA): protocol for a prospective observational study
Source: BMC Rheumatol. 2021 Jun 24;5:23. doi: 10.1186/s41927-021-00187-2 (PMC8223274; doi:10.1186/s41927-021-00187-2)
Supplement: Supplementary file 3 — Additional file 3: Supplement 3. CAP-RA consent form final version 1.423092020 CAP-RA study participant consent form. [file 41927_2021_187_MOESM3_ESM.pdf]

Dedicated to Outstanding care

Sherwood Forest Hospitals NHS Foundation Trust

University of Nottingham UK | CHINA | MALAYSIA

Central Aspects of Pain in Rheumatoid Arthritis (CAP-RA)

IRAS Project ID: 269143 **Participant ID: CAPRA ______ ________ ________**

CONSENT FORM

(Final version 1.4 23/09/2020)

Kindly initial each box as appropriate.

1.	I confirm that I have read the information sheet dated 18/08/2020 (final version 1.2) for the above study. I have had the opportunity to consider the information, ask questions and have had these answered satisfactorily.	☐
2.	I understand that my participation is voluntary and that I am free to withdraw at any time without giving any reason, without my medical care or legal rights being affected. I understand that should I withdraw then the information collected so far cannot be erased and that this information may still be used in the project analysis.	☐
3.	I understand that relevant sections of my medical notes and data collected during the study, may be looked at by authorised individuals from the University of Nottingham, the research group, regulatory authorities or Sherwood Forest Hospitals NHS Foundation Trust, where it is relevant to my taking part in this research. I give permission for these individuals to have access to my records and to collect, store, analyse and publish information obtained from my participation in this study. I understand that my personal details will be kept confidential.	☐
4.	I understand that I would provide a blood sample which researchers may use to measure inflammation and other molecules relevant to arthritis. The sample may be used to conduct DNA genetic analysis.	☐
5.	(**OPTIONAL**) I agree that the samples I have given and the information gathered about me can be stored by the University of Nottingham at the tissue repository, for possible use in future studies. I understand that some of these studies may be carried out by researchers other than the current team who ran the first study, including researchers working for commercial companies. Any samples or data used will be anonymised, and I will not be identified in anyway.	☐
6.	(**OPTIONAL**) I agree to be sent weekly text messages for 12 weeks, asking me to respond to a question about my pain and fatigue levels over each past week.	☐

When completed: 1 for participant; 1 for researcher site file; 1 to be kept in medical notes.

7. (**OPTIONAL**) I understand that researchers at the University of Nottingham might contact me with further information or invite me to contribute further to their research. I understand that receiving further information or being contacted will not commit me to joining any studies. ☐

____________________	____________________	____________________
Name of participant	Date	Signature
____________________	____________________	____________________
Name of person taking consent	Date	Signature
____________________	____________________	____________________
Name of researcher	Date	Signature

3 copies: 1 for participant, 1 for the project file and 1 for the medical notes
